# Design, Synthesis and Biological Evaluation of Brain-Targeted Thiamine Disulfide Prodrugs of Ampakine Compound LCX001

**DOI:** 10.3390/molecules21040488

**Published:** 2016-04-14

**Authors:** Dian Xiao, Fan-Hua Meng, Wei Dai, Zheng Yong, Jin-Qiu Liu, Xin-Bo Zhou, Song Li

**Affiliations:** Laboratory of Computer-Aided Drug Design & Discovery, Beijing Institute of Pharmacology and Toxicology, Beijing 100850, China; be_xiaodian@163.com (D.X.); mengfh331@126.com (F.-H.M); twotou3@126.com (W.D.); yongzhabc@126.com (Z.Y.); fri_autumn@163.com (J.-Q.L.); lis@bmi.ac.cn (S.L.)

**Keywords:** opiate, respiratory depression, ampakines, thiamine disulfide prodrugs, brain distribution

## Abstract

Ampakine compounds have been shown to reverse opiate-induced respiratory depression by activation of amino-3-hydroxy-5-methyl-4-isoxazolepropionic acid (AMPA) glutamate receptors. However, their pharmacological exploitations are hindered by low blood-brain barrier (BBB) permeability and limited brain distribution. Here, we explored whether thiamine disulfide prodrugs with the ability of “lock-in” can be used to solve these problems. A series of thiamine disulfide prodrugs **7a**–**7f** of ampakine compound LCX001 was synthesized and evaluated. The trials *in vitro* showed that prodrugs **7e**, **7d**, **7f** possessed a certain stability in plasma and quickly decomposed in brain homogenate by the disulfide reductase. *In vivo*, prodrug **7e** decreased the peripheral distribution of LCX001 and significantly increased brain distribution of LCX001 after i.v. administration. This compound showed 2.23- and 3.29-fold greater increases in the AUC_0-t_ and MRT_0-t_ of LCX001 in brain, respectively, than did LCX001 itself. A preliminary pharmacodynamic study indicated that the required molar dose of prodrug **7e** was only one eighth that of LCX001 required to achieve the same effect in mice. These findings provide an important reference to evaluate the clinical outlook of ampakine compounds.

## 1. Introduction

The use of opioid analgesics is still the most effective way to manage moderate to severe pain in the clinic [1]. Nevertheless, opiate-induced respiratory depression is a life-threatening condition and a leading cause of death that may arise not only from overdoses, but also during routine procedures supervised by clinicians, including surgical anaesthesia, post-operative analgesia, and as a result of routine out-patient management of pain from cancer, accidents, or other illnesses [2,3,4,5]. Naloxone and related opioid antagonists are currently used to counter respiratory depression in an emergency setting [6]. However, those agents reverse analgesia, so selective antagonism of the respiratory depressive effects of opioids without decreasing their analgesic effects would be a major step toward improving opioid safety.

Recent studies demonstrated that AMPA receptor was a critical component for the generation of respiratory rhythm within the pre-Botzinger complex [7,8,9,10], and was becoming an ideal target for reversing respiratory depression. Ampakine compounds, developed by Cortex Pharmaceuticals, such as CX717, CX1739 and CX1942 *etc*., had been proved to modulate respiratory drive and rhythmogenesis positively by activation of AMPA receptor [11,12,13,14,15,16,17,18]. Most advanced ampakine compounds are CX717 and CX1739 [19], both of which are currently in Phase II clinical trials. Studies demonstrated that a single oral dose of 1500 mg of CX717 had positive effects on respiratory depression induced by pain relieving opiates, without affecting the pain relieving effects [14]. The reversal degree of the basal respiratory rate was similar to that obtained with the opioid antagonist, naloxone. Similar studies in respiratory depression with CX1739 at a high dose of 900 mg have also been planned [20]. 

**Figure 1:**
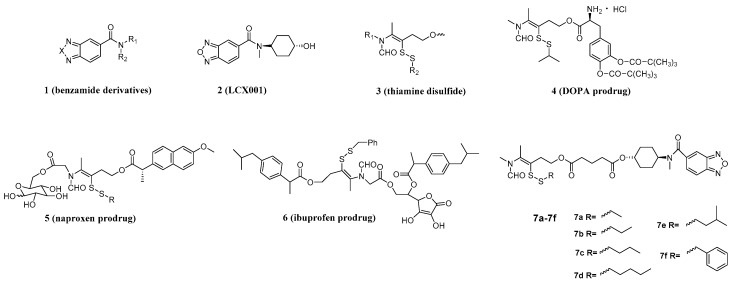
Structure of benzamide derivatives, LCX001, thiamine disulfide, DOPA prodrug, naproxen prodrug, ibuprofen prodrug and the designed prodrugs of LCX001.

Structurally these compounds belong to the benzamide derivative type **1** (Figure 1), but their specific structures remained unknown. According to Cortex’s patents [21,22], we found that compound **2** (Figure 1, LCX001) was one of the most active compounds, with the best electrophysiological effect giving a 21% increase in amplitude of the field excitatory postsynaptic potential (EPSP) in the rat dentate gyrus and producing a 100% inhibition of damphetamine- stimulated locomotion in the behavioral testing [21], so we infered that LCX001 was probably an analogue of the clinical compounds or even one of them. To date, no further developments for these compounds were reported for this indication. We assumed that besides poor absorption the low blood-brain barrier permeability (tPSA of LCX001: 74.49) and limited brain distribution might hinder the development of the compounds. As a result, high-dose use of drugs and non-targeting distribution could cause serious safety problems. In 2007, Food and Drug Administration (FDA) even rejected an investigational new drug (IND) application for CX717 as a treatment for attention-deficit hyperactivity disorder (ADHD) because of its toxicity after chronic dosing at very high dose levels in animals.

**Figure 2:**
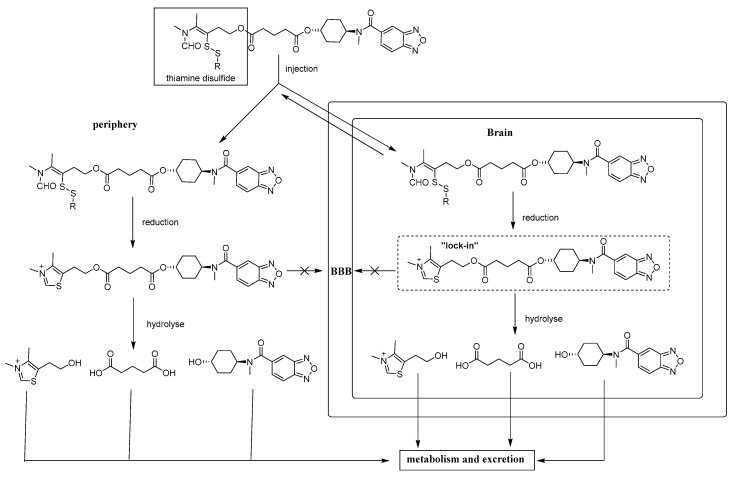
Sequential metabolism and brain “lock-in” pathways of brain-targeted thiamine disulfide prodrugs of LCX001.

The thiamine disulfide system (TDS), has been widely applied as a classic brain-targeted prodrug strategy to a variety of drugs such as DOPA, naproxen, ibuprofen (Figure 1) *etc.* [23,24,25]. In this approach, the increased lipophilic nature of the prodrugs could facilitate passive diffusion across the BBB; subsequently, TDS was reduced and ring-closed to be a thiazolium by disulfide reductase once across the BBB, and then the prodrugs with this system were “locked” in the brain where they can provide sustained release of the active drug via hydrolysis (Figure 2) [23].

In this paper, we introduced the lipophilic TDS with “lock-in” ability to mediate the penetration of LCX001 and designed some brain-targeted prodrugs **7a**–**7f** (Figure 1), which were expected to have synergism and attenuation effects and also provide an important reference value to evaluate the clinical outlook of ampakine compounds. These designed compounds were sequentially synthesized. The *in vitro* stabilities of these prodrugs were discussed. Furthermore, this account also included the biodistribution and pharmacodynamic study of these prodrugs in mice after i.v. administration.

## 2. Results and Discussion

### 2.1. Chemistry

**Scheme 1:**
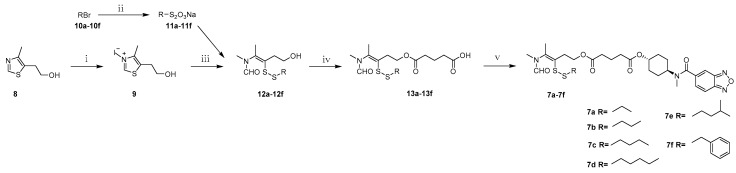
General synthetic route for synthesis of compounds **7a**–**7f**. *Reagents and conditions*: (i) iodomethane, 50 °C, 2 h, 96.2%; (ii) Na_2_S_2_O_3_·5H_2_O , ethanol/H_2_O, 110 °C, 7 h; (iii) NaOH, H_2_O, rt, 1 h, two steps, 13.3%–70.5%; (iv) glutaric anhydride, DMAP, DCM, 50 °C, 5 h; (v) LCX001, DMAP, HOBT, EDCI, TEA, DCM, 50 °C, 5 h, 38.3%–57.5%.

The synthetic routes for the prodrugs **7a**–**7f** are shown in Scheme 1. As shown, *N*,4-dimethyl-5-(2-(hydroxy)ethyl)thiazolium iodide (**9**) can be obtained through methylation of 5-(2-hydroxyethyl)-4-methylthiazole (**8**). Halohydrocarbons **10a**–**10f** and sodium thiosulfate pentahydrate were mixed and refluxed for 7 h to afford intermediates **11a**–**11f**. Thiamine disulfide derivatives **12a**–**12f** were obtained by the reaction of *N*,4-dimethyl-5-(2-(hydroxy)ethyl) thiazolium iodide (**9**) with intermediates **11a**–**11f** in the presence of NaOH at room temperature. Thiamine disulfide derivatives **12a**–**12f** further react with glutaric anhydride to give the requisite intermediate **13a**–**13f**. These compounds were conjugated with LCX001 in the presence of EDCI, HOBT, DMAP and triethylamine to afford the prodrugs **7a**–**7f**. The structures of all the synthesized prodrugs **7a**–**7f** have been confirmed by NMR, and MS, and all the corresponding data can be found in the Experimental Section.

### 2.2. Metabolism Studies of the Prodrugs

#### 2.2.1. Metabolic Stability

To determine the plasma stability and brain stability of these prodrugs, we assayed the pharmacokinetics of the prepared compounds **7a**–**7f** in mice plasma extract and brain homogenate at 37 °C. The pseudo-first order rate constants of disappearance (*K*_e_) of the prodrugs and their half lives (*t*_1/2_) which were calculated by linear regression of Ln of peak area against time in minutes are listed in Table 1.

In plasma, compounds **7a**–**7f** decomposed at a high rate. This instability is due to the existence of ester and disulfide linkages in **7a**–**7f**. The stability of these prodrugs had a descending trend of **7d** (*t*_1/2_ = 15.41 min) > **7c** (*t*_1/2_ = 10.24 min) > **7b** (*t*_1/2_ = 9.82 min) > **7a** (*t*_1/2_ = 2.23 min), indicating that the longer the carbon chain, the better the plasma stability. Larger steric hinderance produced by longer carbon chain hindered the reduction of the disulfide bond. The half-lives of **7d** and **7e** were similar, suggesting that substitution in the side chain had little influence on the plasma stability. We tried to further increase the plasma stability of the compounds by introducing the benzyl group, but no effect was obvious. In spite of a relatively high metabolic rate, it is reasonable to give enough time (*t*_1/2_ = 15.41 min–15.97 min) for the compounds **7d**, **7e**, **7f** to be distributed and reach the brain before complete decomposition.

In brain homogenate, all the compounds decomposed swiftly (*t*_1/2_ = 3.48 min–18.78 min) probably due to the abundance of disulfide reductase in brain. They underwent the expected reduction and cyclization of the thiamine disulfide component to the corresponding thiazolium in brain homogenate. These compounds could be reduced to strongly polar products with high rates, and thus, contributed to the “lock-in” effect in the brain before they were effluxed to the periphery.

#### 2.2.2. Release of the Parent Drug by Prodrugs in Brain Homogenate

**Figure 3:**
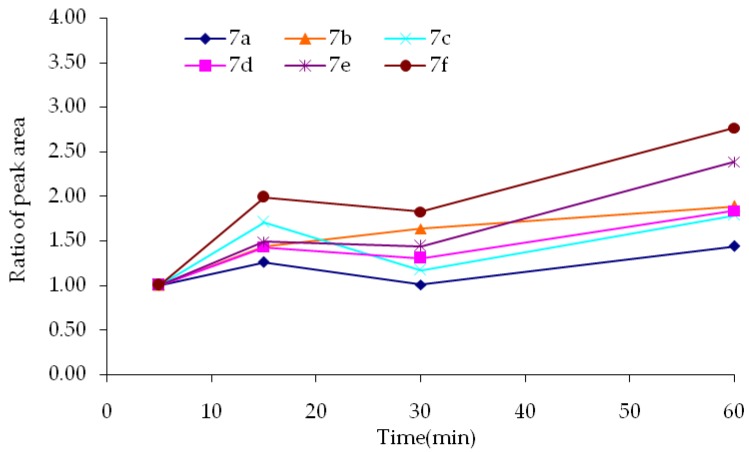
Deliverence of LCX001 by the prodrugs in brain homogenate.

To study the release of LCX001 by the prodrugs in brain, the peak in the LC-MS/MS corresponding to the parent compound was also analyzed. A certain increase in peak area over time was observed, indicating that the prodrugs were indeed converted to the parent drug by esterases in mice brain homogenate. Although the deliverance of LCX001 seemed not fast, the concentration increased steadily as time went by, which might indicate sustained release of LCX001 by the prodrugs in the brain. The release rates of different produgs were similar, which indicated that the release process of parent drug was unlikely to be seriously affected by the formation of a thiazolium cation. Peak integrals were normalized by dividing by the area for the peak of each prodrug itself at 5 min. The ratios are shown in Figure 3.

Several conclusions could be drawn from the above results: first, the half-lives of the prodrugs were mainly influenced by the length of the carbon chains linked to the disulfide functional group. Second, disulfide reductase produced reduction and ring closure of compounds **7a**–**7f** predominated over their clearance in brain with high efficiency, since the decomposition of the prodrugs consisted of reduction and hydrolysis, and the hydrolysis occurs at a relatively low rate. Finally, the release process of the parent drug was unlikely to be seriously affected by the formation of a thiazolium ion. As the above *in vitro* studies demonstrated that prodrugs **7e**, **7d**, **7f** possess preliminary and favorable physicochemical properties, we selected compound **7e** as a model compound to proceed with an *in vivo* study.

### 2.3. In Vivo Studies

#### 2.3.1. Pharmacokinetics in Plasma of the Prodrug **7e**

**Figure 4:**
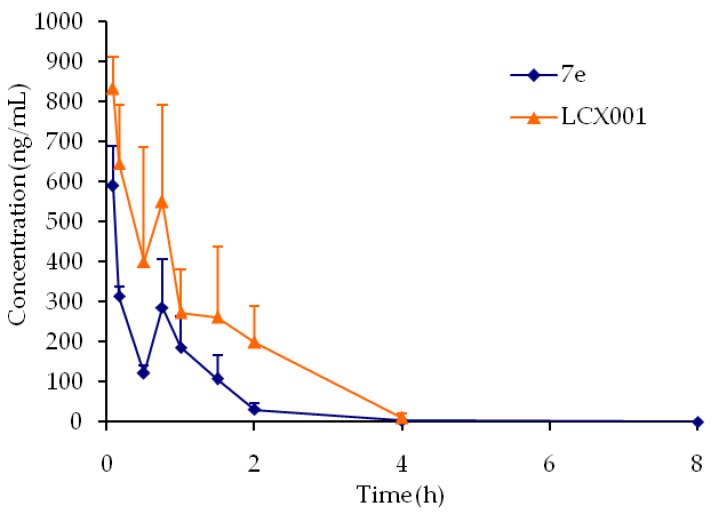
Concentration curve of LCX001 in plasma *versus* time after administration of LCX001 and prodrug **7e**. Error bars show the value of SD (*n* = 3).

To understand the *in vivo* behavior of the prodrug, we assessed the plasma pharmacokinetics of LCX001 and prodrug **7e** in mice (Figure 4).

The compounds were injected through the caudal vein of the mice with a single dose equivalent to 1 mg/kg body weight of LCX001, and then blood was collected to analyze the concentration of LCX001 at different intervals by an LC-MS/MS method. The pharmacokinetic parameters of LCX001 in blood are reported in Table 2.

Compared with the concentration curve of LCX001 after administration of underivatized LCX001, prodrug **7e** exhibited significantly lower concentrations at different time intervals. Free LCX001 of prodrug **7e** and LCX001 presented with an area under the concentration-time profile (AUC_0-t_) ratio of 0.40 and the mean residence times (MRT) of LCX001 in plasma after i.v. administration of **7e** was 0.77 times that of LCX001, indicating that **7e** could increase brain distribution because of its increased lipophilicity and be cleared more quickly than LCX001 due to the strongly polar thiazolium cation produced by reduction of the corresponding thiamine disulfides.

#### 2.3.2. Brain Distribution of Prodrug **7e**

**Figure 5:**
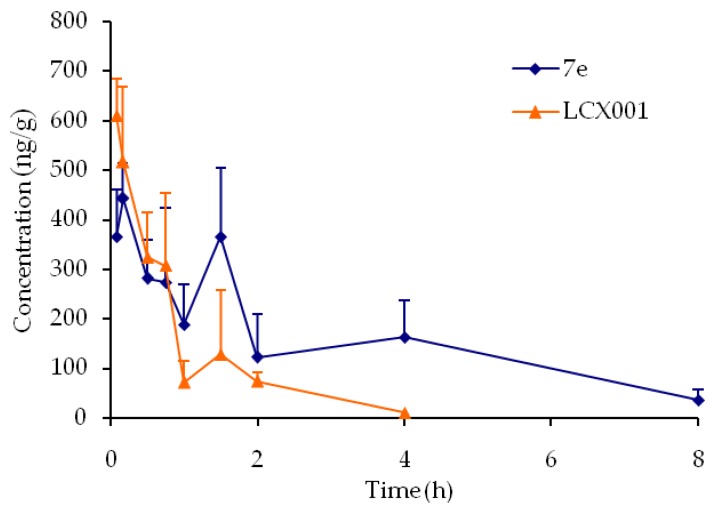
Concentration curve of LCX001 in brain *versus* time after administration of LCX001 and prodrug **7e**. Error bars show the value of SD (*n* = 3).

To further evaluate the possibility of prodrug **7e** being transported across the BBB, the brain distribution of prodrug **7e** and LCX001 after injection were detected at fixed time intervals. The concentrations of LCX001 in brain *versus* time curves are displayed in Figure 5. The curves show that prodrug **7e** exhibited much higher distribution in brain. The pharmacokinetic parameters of LCX001 in brain are reported in Table 3. The data showed that *C*_max_ of LCX001 in brain after i.v. administration of prodrug **7e** was not significantly improved, but the AUC_0-t_ and MRT_0-t_ of LCX001 was significantly higher than that after the injection of the parent compound LCX001. The AUC_0-t_ for prodrug **7e** was enhanced to 2.23 times that of LCX001. The MRT_0-t_ was increased to 3.29 times that of LCX001. The curves displayed that LCX001 could be quickly metabolized or effluxed while prodrug **7e** showed a certain stability. This agreed well with the fact that the “lock-in” effect and sustained release effect of the prodrug **7e** could effectively delay the clearance of LCX001.

### 2.4. Pharmacodynamic Study of Prodrug ***7e***

To investigate the effect of **7e** on opiate-induced respiratory depression, we proceeded with a preliminary pharmacodynamic study in mice. The potent opiate receptor agonist 030418 (K_i_ = 0.91 nM for μ-opioid receptor) [26] was administered to induce fatal apneas. Varying doses of prodrug **7e** were administered 10 min before 030418 administration. The effect of prodrug **7e** and LCX001 on opiate-induced respiratory depression was evaluted by the mortality of mice. The results are reported in Table 4. The data suggested that prodrug **7e** reduced the death rate in a dose-dependent manner. Moreover, 3 mg/kg prodrug **7e** gave the same effect as 10 mg/kg LCX001 on reducing mortality, which indicated that the required molar dose of **7e** was only one eighth of that of LCX001 to achieve the same effect. Further pharmacodynamic studies are currently underway.

## 3. Experimental Section

### 3.1. General Information

TLC was performed using precoated silica gel plates (Yantai dexin Bio-Technology Co., Ltd., Yantai, China). Column chromatography was performed using silica gel (200–300 mesh; Yantai Chemical Industry Research Institute, Yantai, China). Melting points were measured on a RY-1 apparatus (Tianda Tianfa Technology Co., Ltd., Tianjin, China). NMR spectra were recorded on a JNM-ECA-400 400 MHz spectrometer (JEOL Ltd., Tokyo, Japan) using CDCl_3_, DMSO-d_6_ and D_2_O as solvent. Chemical shifts are expressed in δ (ppm), with tetramethylsilane (TMS) functioning as the internal reference, coupling constants (*J*) were expressed in Hz. Mass spectroscopy (MS) was carried out on an API 3000 triple-quadrupole mass spectrometer (AB Sciex, Concord, ON, Canada) equipped with a Turbo IonSpray electrospray ionization (ESI) source was used for mass analysis and detection. Analyst 1.4 software (AB Sciex) was used for data acquisition.

### 3.2. Liquid Chromatographic Conditions

An Agilent 1100 system (Agilent Technologies, Palo Alto, CA, USA) consisting of a vacuum degasser, a quaternary pump, and an autosampler was used for HPLC solvent and sample delivery. Chromatographic separation was achieved using a Poroshell 120 C_18_ column (50 mm × 2.1 mm, 2.7 μm, Agilent) and a C_18_ guard column (4 × 3.0 mm, 5 μm; Phenomenex, Torrance, CA, USA). The column temperature was maintained at 25 °C. Gradient elution was performed using purified water as solvent A and acetonitrile as solvent B. Two elution gradient programs were used. The program applied for *in vitro* samples was as follows: 0.0–0.5 min 10% B to 80% B; 0.5–1.5 min 80% B; 1.5–1.6 min 80% B to 10% B; 1.6–6.0 min 10% B. And the program applied for *in vivo* samples was as follows: 0.0–0.5 min 10% B to 80% B; 0.5–1.3 min 80% B; 1.3–1.5 min 80% B to 10% B; 1.5–7.0 min 10% B. The flow rate was set at 0.3 mL/min. The injection volume was 10 μL.

### 3.3. Mass Spectrometric Conditions

The mass spectrometer was operated in the positive-ion mode. Ultrapure nitrogen was used as a nebulizer, curtain, and collision-activated dissociation (CAD) gas at 8, 10, and 6 instrument units, respectively. The optimized Turbo IonSpray voltage and temperature were set to 4500 V and 450 °C, respectively. Quantification was performed with multiple reaction monitoring. Glipizide was chosen as internal standard. The transitions and the optimized collision energies (CE) for each compound were listed in Table 5. Each transition was monitored with a 150-ms dwell-time.

### 3.4. Stability in Plasma Extract and Brain Homogenate

Blood was drawn from mice though the orbital sinus and was collected in a heparinized tube paved with heparin sodium. Samples were centrifuged at 10,000 rpm for 16 min to separate plasma which was diluted with four volumes of water. The brain was removed and homogenized. Samples were then placed on ice and used immediately. Ten μL acetonitrile solution (10 μg/mL) of the compound was added to 50 μL of plasma or brain homogenate and gently vortexed. Samples were incubated at 37 °C and aliquots were removed after 0, 5, 15, 30, 60 and 90 min, respectively. One hundred μL acetonitrile was added to each and vortexed. Samples were centrifuged for 10 min to remove proteins and the supernatants were analyzed by LC-MS/MS method as described.

### 3.5. Release of the Parent Drug by Prodrugs in Brain Homogenate

The prodrugs were treated in brain homogenate according to the procedure in Section 3.4 except that the intervals were changed to 0, 5, 15, 30 and 60 min. The peak of LCX001 was analyzed by LC-MS/MS described in Section 3.2 and Section 3.3. Peak integrals were normalized by dividing by the area for the peak of each prodrug itself at 5 min.

### 3.6. Biodistribution Studies in Vivo

#### 3.6.1. Test Animals

Adult Kunming mice weighing 20–22 g were obtained from the animal center of academy of military medical sciences. The animals were left for two days to acclimatize to animal room conditions and were maintained on standard pellet diet and water *ad libitum*. Food was withdrawn on the day before the experiment, but free access to water was allowed. Since the experiments could be completed within 24 h, there was no significant change in the mice body weight during the experiment. All animals received human care, and the study protocols complied with the guidelines of the animal center of academy of military medical sciences. Throughout the experiments, the animals were handled according to the international ethical guidelines for the care of laboratory animals. 

#### 3.6.2. Biodistribution Studies of the Prodrug and LCX001

Mice were randomly divided into two groups, 27 in each group, for different sampling times and housed in one cage. Each animal was injected with **7e** or LCX001 in 20% *w*/*v* hydroxypropyl-β-cyclodextrin and saline (0.45%) solution through the tail vein at a single dose equivalent to 1 mg/kg body weight of LCX001. At appropriate time intervals (5, 10, 30, 45, 60, 90, 120, 240 and 480 min), the animals were sacrificed and 1 mL blood samples withdrawn from the orbital sinus were collected in heparinized tubes. Fifty μL plasma was immediately separated by centrifugation, diluted with 200 μL methanol and stored at −20 °C until assay. Meanwhile, the brain was removed, weighed, homogenized and diluted with methanol to 1:5 (g/mL). The homogenates were also stored at −20 °C until assay. Internal standard (200 ng/mL) was added to the samples. The samples were vortexed for 3 min and then centrifuged at 9500 rpm for 15 min. Fifty μL supernatants were withdrawn, then 50 μL of water was added to each aliquot and vortexed. Ten μL samples were analyzed by LC-MS/MS method as described.

### 3.7. Statistical Analysis

The *C*_max_, AUC_0-t_, *T*_max_, MRT_0-t_ and *t*_1/2_ were calculated by WinNonlin software (version 5.2.1, Pharsight, Mountain View, CA, USA). Statistical evaluation was performed using analysis of variance followed by *t-*test. A value of *p* < 0.05 was considered significant.

### 3.8. Pharmacodynamic Study of Prodrug ***7e***

#### 3.8.1. Test Animals

A total of 80 male Kunming mice weighing 20–22 g were used in this study. Animals were fed *ad libitum* with a commercial rodent feed and had free access to drinking water. All animals received human care, and the study protocols complied with the guidelines of the animal center of academy of military medical sciences. Throughout the experiments, the animals were handled according to the international ethical guidelines for the care of laboratory animals. 

#### 3.8.2. Model of Opiate-Induced Respiratory Depression

Respiratory depression in conscious mice was induced by hypodermic (15 mg/kg) administration of 030418. Ten min prior to 030418 administration, animals were randomly assigned to the experimental groups and received an intravenous injection of either saline solution (control), LCX001 (10 mg/kg), prodrug **7e** (30 mg/kg, 10 mg/kg, 3 mg/kg, 1 mg/kg or 0.5 mg/kg) (Table 4). 

#### 3.8.3. Assessment of the Prodrug **7e** Effect

During the experiment, the poisoning time, poisoning symptoms and death time were recorded. The death rate (death number/sample number) was used to evaluate the effect of **7e** on opiate-induced respiratory depression.

### 3.9. Synthesis

*N,4-Dimethyl-5-(2-(hydroxy)ethyl)thiazolium iodide* (**9**). 5-(2-Hydroxyethyl)-4-methylthiazole (100.0 g, 698.2 mmol) and methyl iodide (100.0 mL, 1500.0 mmol) were mixed and refluxed for 2 h. After evaporation of excess methyl iodide, to the residual brown syrup was added ether (100 mL) and stirred for 30 min, the precipitate was filtered to yield **9** as a pale-yellow solid (191.4 g, 96.2%). Mp: 82–84 °C. ^1^H-NMR (DMSO-*d*_6_, ppm): 2.43 (s, 3H), 3.03 (t, 2H, *J* = 5.5 Hz), 3.63 (t, 2H, *J* = 5.6 Hz), 4.09 (s, 3H), 9.96 (s, 1H); MS *m*/*z* [M]^+^ calculated for C_7_H_12_NS^+^: 158.1; found: 158.1.

*General procedure for synthesis of S-substituted sodium thiosulfates* **11a**–**11f**. A solution of sodium thiosulfate pentahydrate (24.8 g, 100.0 mmol) in water (60 mL) was added to the solution of alkyl bromide (100 mmol) in ethanol (30 mL) under stirring. The reation mixture was heated to 110 °C and maintained for 7 h. After evaporation of the solvent, the residual white solid was dried in a vacuum desiccator to yield *S*-substituted sodium thiosulfates **11a**–**11f**.

*General procedure for synthesis of compounds* **12a**–**12f**. Under argon protection, to a 30 mL aqueous of quaternary ammonium salt **9** (14.3 g, 50.0 mmol) and sodium hydroxide (4.0 g, 100.0 mmol) was added *S*-substituted sodium thiosulfates **11a**–**11f** (120.0 mmol). The reaction mixture was stirred for 1 h at room temperature. The resultant oily substance was extracted with ethyl acetate (450 mL) and the organic layer was dried over anhydrous Na_2_SO_4_ and concentrated under reduced pressure. Purification of the crude product by column chromatography with dichloromethane-methanol (100:1) as eluent gave a yellow oil.

*N-(3-(Ethyldisulfanyl)-5-hydroxypent-2-en-2-yl)-N-methylformamide* (**12a**, Figure S1). 40.9% yield as a yellow oil. ^1^H-NMR (CDCl_3_, ppm): 1.19 (t, 3H, *J* = 7.3 Hz), 1.96, 1.86 (2s, 3H), 2.64 (q, 2H, *J* = 7.3 Hz), 2.70 (t, 2H, *J* = 6.7 Hz), 2.98, 2.83 (2s, 3H), 3.55–3.43 (m, 2H), 7.96, 7.84 (2s, 1H). MS *m*/*z* [M + H]^+^ calculated for C_9_H_17_NO_2_S_2_: 236.1; found: 236.2.

*N-(3-(Propyldisulfanyl)-5-hydroxypent-2-en-2-yl)-N-methylformamide* (**12b**, Figure S2). 64.8% yield as a yellow oil. ^1^H-NMR (CDCl_3_, ppm): 0.97 (t, 3H, *J* = 7.3 Hz), 1.69–1.63 (m, 2H), 2.01, 1.96 (2s, 3H), 2.47 (br, 1H), 2.59 (t, 2H, *J* = 7.6 Hz), 2.87 (t, 2H, *J* = 6.7 Hz), 3.06, 2.95 (2s, 3H), 3.81 (t, 3H, *J* = 6.4 Hz), 7.96, 7.92 (2s, 1H); MS *m*/*z* [M + H]^+^ calculated for C_10_H_19_NO_2_S_2_: 250.1; found: 250.1.

*N-(3-(Butyldisulfanyl)-5-hydroxypent-2-en-2-yl)-N-methylformamide* (**12c**, Figure S3). 70.5% yield as a yellow oil. ^1^H-NMR (CDCl_3_, ppm): 0.91(t, 3H, *J* = 7.3 Hz), 1.43–1.33 (m, 2H), 1.65–1.57 (m, 2H), 2.01, 1.96 (2s, 3H), 2.51 (br, 1H), 2.62 (t, 2H, *J* = 7.3 Hz), 2.87 (t, 2H, *J* = 6.7 Hz), 3.06, 2.95 (2s, 3H), 3.81 (t, 2H, *J* = 6.8 Hz), 7.96, 7.92 (2s, 1H). MS *m*/*z* [M + H]^+^ calculated for C_11_H_21_NO_2_S_2_: 264.1; found: 264.2.

*N-(3-(Amyldisulfanyl)-5-hydroxypent-2-en-2-yl)-N-methylformamide* (**12d**, Figure S4). 52.0% yield as a yellow oil. ^1^H-NMR (CDCl_3_, ppm): 0.90 (t, 3H, *J* = 7.3 Hz), 1.36–1.29 (m, 4H), 1.65–1.60 (m, 2H), 2.01, 1.97 (2s, 3H), 2.49 (br, 1H), 2.61 (t, 2H, *J* = 7.3 Hz), 2.87 (t, 2H, *J* = 6.7 Hz), 3.06, 2.95 (2s, 3H), 3.80 (t, 2H, *J* = 6.8 Hz), 7.96, 7.92 (2s, 1H). MS *m*/*z* [M + H]^+^ calculated for C_12_H_23_NO_2_S_2_: 278.1; found: 278.0.

*N-(3-Isoamyldisulfanyl)-5-hydroxypent-2-en-2-yl)-N-methylformamide* (**12e**, Figure S5). 76.8% yield as a yellow oil. ^1^H-NMR (CDCl_3_, ppm): 1.19 (t, 3H, *J* = 7.3 Hz), 1.53–1.47 (m, 2H), 1.68–1.61 (m, 1H), 2.01, 1.96 (2s, 3H), 2.49 (br, 1H), 2.62 (t, 2H, *J* = 7.6 Hz), 2.88 (t, 2H, *J* = 6.8 Hz), 3.06, 2.95 (2s, 3H), 3.80(t, 3H, *J* = 6.4 Hz), 7.96, 7.92(2s, 3H). MS *m*/*z* [M + H]^+^ calculated for C_12_H_23_NO_2_S_2_: 278.1; found: 278.0.

*N-(3-(Benzyldisulfanyl)-5-hydroxypent-2-en-2-yl)-N-methylformamide* (**12f**, Figure S6). 44.1% yield as a yellow oil. ^1^H-NMR (*d*_6_-DMSO, ppm): 1.91, 1.85 (2s, 3H), 2.62 (t, 2H, *J* = 6.7 Hz), 2.82, 2.81 (2s, 3H), 3.52–3.47 (m, 2H), 3.92 (s, 2H), 4.74–4.70 (m, 1H), 7.35–7.25 (m, 5H), 7.96, 7.81 (2s, 1H). MS *m*/*z* [M + H]^+^ calculated for C_14_H_19_NO_2_S_2_: 298.1; found: 298.2.

*General procedure for synthesis of 4-(N-methylformamido)pent-3-en-1-yl)oxy)-5-oxopentanoic acids* **13a**–**13f**. A stirred solution of intermediate **12a**–**12f** (10.0 mmol) in dry dichloromethane (50 mL) was treated with DMAP (0.1 g, 0.8 mmol) and glutaric anhydride (3.4 g, 30.0 mmol). The mixture was refluxed for 5 h. The solvent was evaporated under vacuum and purification of the crude product by column chromatography with dichloromethane-methanol (100:1) as eluent gave **13a**–**13f** as yellow syrup.

*General procedure for synthesis of compounds* **7a**–**7f**. The intermediate **13a–13f** (4.7 mmol), LCX001 (1.0 g, 3.6 mmol), triethylamine (0.5 mL, 3.6 mmol) and HOBT (486 mg, 3.6 mmol) were dissolved in dry dichloromethane (50 mL) in a round-bottomed flask under string for 10 min. After addition of DMAP (44 mg, 0.36 mmol) and EDCI (1.1 g, 5.4 mmol), the mixture was refluxed for 5 h. The resulting mixture was concentrated by a rotary evaporator to afford yellow oleosus residue that was purified by column chromatography on silica gel (ethyl acetate/petroleum ether, (1:1)) to provide **7a**–**7f** as white solids.

*S-3-(Ethyldisulfanyl)-4-(N-methylformamido)pent-3-en-1-yl((1R,4R)-4-(N-methylbenzo[c][1,2,5]oxadiazole-5-carboxamido)cyclohexyl) glutarate* (**7a**, Figure S7). 43.2% yield as a white solid. Mp: 78.8–79.5 °C; ^1^H-NMR (CDCl_3_, ppm): 1.27 (t, 3H, *J* = 7.3 Hz), 1.99, 1.97 (2s, 3H), 1.94–1.62 (m, 8H), 2.12–1.99 (m, 2H), 2.44–2.35 (m, 4H), 2.63 (q, 2H, *J* = 7.3 Hz), 3.04–2.87 (m, 8H), 4.70–4.59, 3.51 (m, 1H), 4.24 (t, 2H, *J* = 6.7 Hz), 7.41 (d, 1H, *J* = 8.1 Hz); ^13^C-NMR (CDCl_3_, ppm): 172.6, 172.2, 168.6, 162.3, 161.2, 148.4, 139.7, 136.5, 130.6, 117.6, 114.5, 113.6, 71.8, 62.0, 51.9, 33.3, 33.0, 31.8, 30.3, 29.6, 29.6, 29.0, 29.0, 28.2, 26.9, 19.8, 18.4, 14.0. HRMS (ESI+) *m*/*z* [M + H]^+^ calculated for C_28_H_38_N_4_O_7_S_2_: 607.2182; found: 607.2255.

*S-3-(Propyldisulfanyl)-4-(N-methylformamido)pent-3-en-1-yl((1R,4R)-4-(N-methylbenzo[c][1,2,5]oxadiazole-5-carboxamido)cyclohexyl) glutarate* (**7b**, Figure S8). 40.7% yield as a white solid. Mp: 78.0–79.3 °C; ^1^H-NMR (CDCl_3_, ppm): 0.96 (t, 3H, *J* = 7.3 Hz), 1.94–1.23 (m, 10H), 2.00, 1.97 (2s, 3H), 2.13–2.11 (m, 2H), 2.42–2.26 (m, 4H), 2.58 (t, 2H, *J* = 6.2 Hz), 3.04–2.88 (m, 8H), 4.70–4.56, 3.50 (m, 1H), 4.25 (t, 2H, *J* = 5.6 Hz), 7.42 (d, 1H, *J* = 8.9 Hz), 7.98–7.83 (m, 3H); ^13^C-NMR (CDCl_3_, ppm): 172.7, 172.3, 168.8, 162.3, 161.2, 148.4, 139.7, 136.6, 130.7, 117.5, 114.6, 113.5, 71.8, 62.1, 51.9, 41.1, 33.3, 33.1, 30.4, 29.6, 29.6, 29.0, 29.0, 28.2, 26.9, 21.9, 19.9, 18.5, 12.9. HRMS (ESI+) *m*/*z* [M + H]^+^ calculated for C_29_H_40_N_4_O_7_S_2_: 621.2338; found: 621.2411.

*S-3-(Butyldisulfanyl)-4-(N-methylformamido)pent-3-en-1-yl((1R,4R)-4-(N-methylbenzo[c][1,2,5]oxadiazole-5-carboxamido)cyclohexyl) glutarate* (**7c**, Figure S9). 38.3% yield as a white solid. Mp: 67.6–68.4 °C; ^1^H-NMR (CDCl_3_, ppm): 0.91 (t, 3H, *J* = 7.6 Hz), 2.00–1.25 (m, 12H), 2.03, 2.00 (2s, 3H), 2.15–2.09 (m, 2H), 2.40–2.26 (m, 4H), 2.61 (t, 2H, *J* = 7.3 Hz), 3.03–2.87 (m, 8H), 4.72–4.53, 3.51 (m, 1H), 4.25 (t, 2H, J = 7.0 Hz), 7.42 (d, 1H, *J* = 8.7 Hz), 7.94–7.83 (m, 3H); ^13^C-NMR (CDCl_3_, ppm): 172.7, 172.3, 168.6, 162.4, 161.2, 148.3, 139.8 136.6, 130.7, 117.5, 114.6, 113.7, 71.6, 62.1, 52.0, 39.0, 33.4, 33.3, 31,7, 30.4, 29.6, 29.6, 29.0, 29.0, 28.3, 26.9, 21.5, 20.0, 18.5, 13.6. HRMS (ESI+) *m*/*z* [M + H]^+^ calculated for C_30_H_42_N_4_O_7_S_2_: 635.2495; found: 635.2568.

*S-3-(Amyldisulfanyl)-4-(N-methylformamido)pent-3-en-1-yl((1R,4R)-4-(N-methylbenzo[c][1,2,5]oxadiazole-5-carboxamido)cyclohexyl) glutarate* (**7d**, Figure S10). 45.5% yield as a white solid. Mp: 69.3–70.3 °C; ^1^H-NMR (CDCl_3_, ppm): 0.89 (t, 3H, *J* = 6.8 Hz), 1.94–1.28 (m, 14H), 2.00, 1.97 (2s, 3H), 2.14–2.09 (m, 2H), 2.41–2.27 (m, 4H), 2.60 (t, 2H, *J* = 5.8 Hz), 3.04–2.88 (m, 8H), 4.71–4.59, 3.50 (m, 1H), 4.24 (t, 2H, *J* = 6.8 Hz), 7.42 (d, 1H, *J* = 8.4 Hz), 7.94–7.84 (m, 3H); ^13^C-NMR (CDCl_3_, ppm): 172.7, 172.2, 168.8, 162.4, 161.2, 148.5, 139.8, 136.6, 130.4, 117.4, 114.6, 113.7, 71.9, 62.1, 51.9, 39.3, 33.4, 33.1, 31.9, 30.5, 30.2, 29.6, 29.6, 29.0, 29.0, 28.3, 26.9, 22.2, 19.2, 18.5, 13.9. HRMS (ESI+) *m*/*z* [M + H]^+^ calculated for C_31_H_44_N_4_O_7_S_2_: 649.2651; found: 649.2724.

*S-3-(Isoamyldisulfanyl)-4-(N-methylformamido)pent-3-en-1-yl((1R,4R)-4-(N-methylbenzo[c][1,2,5]oxadiazole-5-carboxamido)cyclohexyl) glutarate* (**7e**, Figure S11). 45.0% yield as a white solid. Mp: 74.0–75.4 °C; ^1^H-NMR (CDCl_3_, ppm): 0.89 (d, 6H, *J* = 6.5 Hz), 1.97–1.47 (m, 11H), 2.00,1.97 (2s, 3H), 2.15–2.09 (m, 2H), 2.41–2.26 (m, 4H), 2.62 (t, 2H, *J* = 6.7 Hz), 3.04–2.88 (m, 8H), 4.71–4.57, 3.51 (m, 2H), 4.24 (d, 2H, *J* = 6.4 Hz), 7.42 (d, 1H, *J* = 8.1 Hz), 9.97–7.83 (m, 3H); ^13^C-NMR (CDCl_3_, ppm): 172.7, 172.3, 168.6, 162.4, 161.3, 148.5, 139.7, 136.5, 130.8, 117.4, 114.6, 113.6, 71.8, 62.1, 51.9, 37.6, 37.4, 33.4, 33.1, 31.9, 30.2, 29.6, 29.6, 29.0, 29.0, 28.3, 28.3, 26.9, 22.2, 20.0, 18.5. HRMS (ESI+) *m*/*z* [M + H]^+^ calculated for C_31_H_44_N_4_O_7_S_2_: 649.2651; found: 649.2723.

*S-3-(Benzyldisulfanyl)-4-(N-methylformamido)pent-3-en-1-yl((1R,4R)-4-(N-methylbenzo[c][1,2,5]oxadiazole-5-carboxamido)cyclohexyl) glutarate* (**7f**, Figure S12). 57.5% yield as a white solid. Mp: 72.9–73.8 °C; ^1^H-NMR (CDCl_3_, ppm): 1.91–1.25 (m, 8H), 1.94, 1.91 (2s, 3H), 2.10–2.03 (m, 2H), 2.41–2.25 (m, 4H), 3.00–2.75 (m, 8H), 4.69–4.59, 3.50 (m, 1H), 3.86 (s, 2H), 4.15 (t, 2H, *J* = 7.1 Hz), 7.42–7.28 (m, 5H), 7.97–7.83 (m, 3H). ^13^C-NMR (CDCl_3_, ppm): 172.6, 172.3, 168.8, 162.4, 161.3, 148.4, 139.7, 136.1, 135.8, 130.4, 129.2, 129.2, 128.5, 128.5, 127.7, 117.7, 114.6, 113.6, 71.8, 62.0, 51.9, 44.2, 33.3, 33.3, 31.9, 30.4, 29.5, 29.5, 28.7, 28.7, 26.9, 19.9, 18.4. HRMS (ESI+) *m*/*z* [M + H]^+^ calculated for C_33_H_40_N_4_O_7_S_2_: 669.2338; found: 669.2411.

## 4. Conclusions

In this paper, a series of brain-targeted prodrugs of LCX001 was synthesized and evaluated. The *in vitro* trials showed that prodrugs **7e**, **7d**, **7f** possessed a certain stability in plasma which was propitious to get sufficient time to be delivered across the BBB. Moreover, prodrugs **7e**, **7d**, **7f** were quickly decomposed in brain homogenate by the disulfide reductase, indicating the “lock-in” effect of these compounds after they entered the central nervous system (CNS). *In vivo*, prodrug **7e** decreased the peripheral distribution of LCX001 and significantly increased brain distribution of LCX001 after i.v. administration. As a result, a lower dosage of prodrug **7e** could give the same effect as the parent drug LCX001 on reversing opiate-induced respiratory depression, which was helpful in promoting the therapeutic efficacy and safety. More significantly, this prodrug also provides an important reference value to evaluate the clinical outlook of ampakine compounds.

## Figures and Tables

**Table 1:** Metabolic stability of compounds **7a**–**7f** in mice plasma extract and brain homogenate at 37 °C. 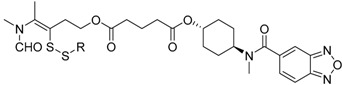

Compound	R	Biological Matrix	Kinetic Constants	
			*K*_e_ (min^−1^) (×10^−2^)	*t*_1/2_ (min)
**7a**	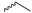	Plasma	31.1	2.23
		Brain	19.9	3.48
**7b**	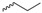	Plasma	7.06	9.82
		Brain	6.82	10.17
**7c**	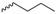	Plasma	6.77	10.24
		Brain	4.05	17.10
**7d**	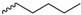	Plasma	4.50	15.41
		Brain	6.34	10.93
**7e**	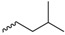	Plasma	4.34	15.97
		Brain	3.69	18.78
**7f**	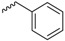	Plasma	4.48	15.47
		Brain	5.78	11.99

**Table 2:** Pharmacokinetic parameters of LCX001 in plasma after administration of LCX001 and prodrug **7e** (*n* = 3).

Compound	*C*_max_ (ng/mL)	AUC_0-t_ (h·ng/mL)	*T*_max_ (h)	*t*_1/2_ (h)	MRT_0-t_ (h)
LCX001	833.67 ± 79.85	951.66 ± 115.52	0.08	0.53 ± 0.17	1.08 ± 0.04
**7e**	590.36 ± 99.90 *	384.16 ± 39.21 *	0.08	0.48 ± 0.20	0.83 ± 0.03 *

* *p* < 0.05 with respect to naked LCX001.

**Table 3:** Pharmacokinetic parameters of LCX001 in brain after administration of LCX001 and prodrug **7e** (*n* = 3).

Compound	*C*_max_ (ng/g)	AUC_0-t_ (h·ng/g)	*T*_max_ (h)	*t*_1/2_ (h)	MRT_0-t_ (h)
LCX001	609.40 ± 73.66	504.32 ± 204.71	0.08	0.59 ± 0.08	0.70 ± 0.22
**7e**	454.53 ± 74.69	1125.49 ± 336.57 *	0.17	2.50 ± 0.42 *	2.30 ± 0.73 *

* *p* < 0.05 with respect to naked LCX001.

**Table 4:** Mortality of opiate poisoned mice after administration of LCX001 and prodrug **7e**.

Experiment	Treatment	Test Material	*n*	ROUTE	Dose (mg/kg)	Death Number (*n*)	Death Rate
RD	030418, 15 mg/kg, sc	Saline	10	iv		8	80%
RD	030418, 15 mg/kg, sc	LCX001	10	iv	10	2	20%
RD	030418, 15 mg/kg, sc	**7e**	10	iv	30	0	0%
RD	030418, 15 mg/kg, sc	**7e**	10	iv	10	0	0%
RD	030418, 15 mg/kg, sc	**7e**	20	iv	3	4	20%
RD	030418, 15 mg/kg, sc	**7e**	10	iv	1	3	30%
RD	030418, 15 mg/kg, sc	**7e**	10	iv	0.5	5	50%

RD—Respiratory depression; sc—subcutaneous injection, iv—intravenous injection.

**Table 5:** Mass spectrometric conditions for the compounds.

[M + H]^+^	Precursor ion	Product ion	CE (V)
LCX001	276.2	178.2	20
Glipizide	446.0	321.0	20
**7a**	607.5	258.2	25
**7b**	621.5	258.2	26
**7c**	635.5	258.2	27
**7d**	649.5	258.2	29
**7e**	649.5	258.2	29
**7f**	669.5	258.2	29

## Supplementary Materials

Supplementary materials can be accessed at: http://www.mdpi.com/1420-3049/21/4/488/s1.

## Abbreviations

The following abbreviations are used in this manuscript:

AMPA	Amino-3-hydroxy-5-methyl-4-isoxazolepropionic acid
BBB	Blood-brain barrier
EPSP	Excitatory postsynaptic potential
FDA	Food and Drug Administration
IND	Investigational new drug
ADHD	Attention-deficit hyperactivity disorder
TDS	Thiamine disulfide system
EDCI	1-Ethyl-3-(3-dimethylaminopropyl)carbodiimide hydrochloride
HOBT	*N*-Hydroxybenzotriazole
DMAP	4-Dimethylaminopyridine
TEA	Triethylamine
DCM	Dichloromethane
rt	Room temperature
MRT	Mean residence times
RD	Respiratory depression
sc	Subcutaneous injection
iv	Intravenous injection
NMR	Nuclear Magnetic Resonance
LC	Liquid chromatography
MS	Mass spectrometry
ESI	Electrospray ionization
CAD	Collision-activated dissociation
CE	Collision energies
USA	United States of America
TMS	Tetramethylsilane
