# Supplementary material for: Design, Synthesis and Biological Evaluation of Brain-Targeted Thiamine Disulfide Prodrugs of Ampakine Compound LCX001
Source: Molecules. 2016 Apr 14;21(4):488. doi: 10.3390/molecules21040488 (PMC6274124; doi:10.3390/molecules21040488)
Supplement: Supplementary file 1 [file molecules-21-00488-s001.pdf]

Supplementary Materials: Design, Synthesis and Biological Evaluation of Brain-Targeted Thiamine Disulfide Prodrugs of Ampakine Compound LCX001

Dian Xiao, Fan-Hua Meng, Wei Dai, Zheng Yong, Jin-Qiu Liu, Xin-Bo Zhou and Song Li

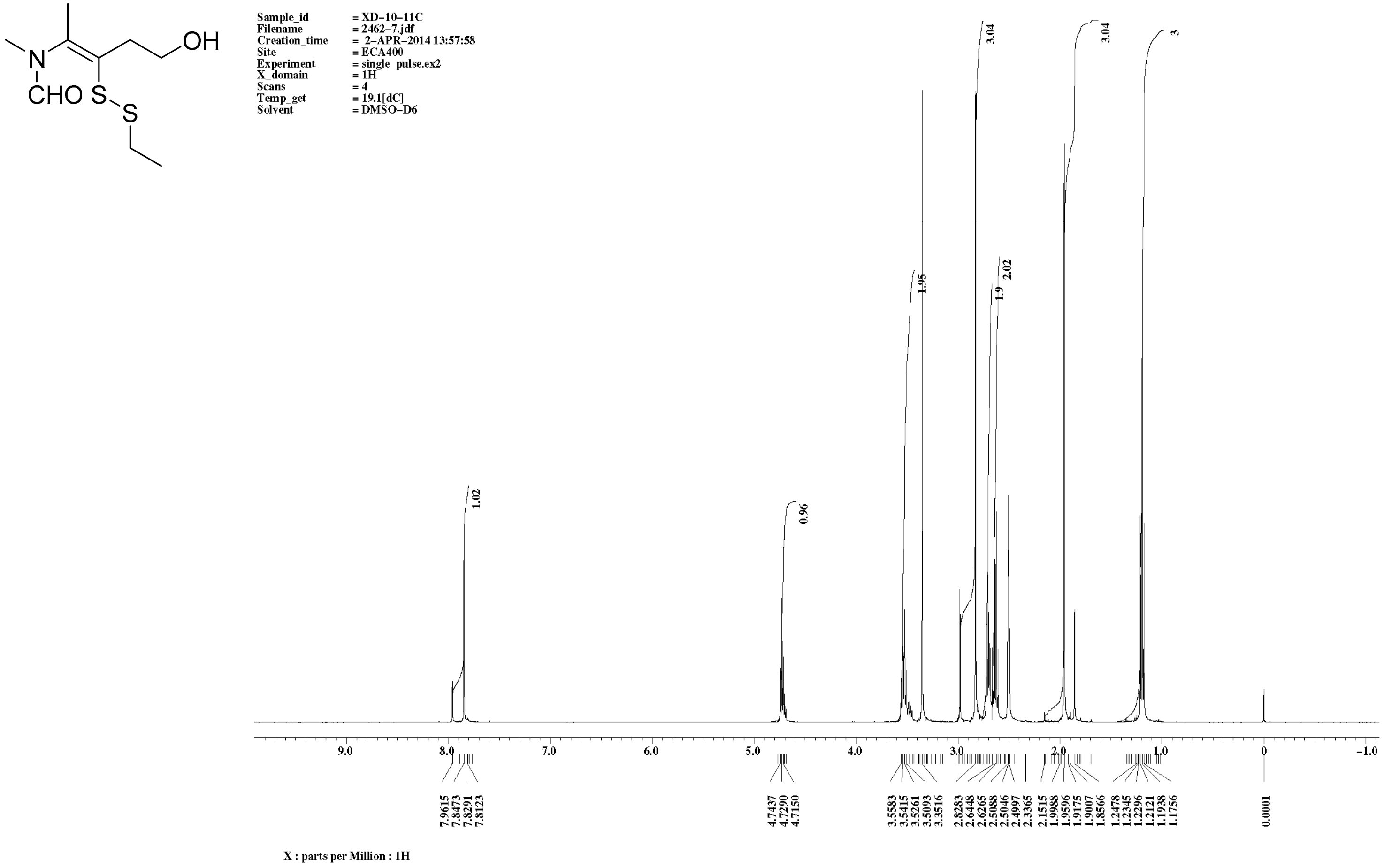

Figure S1. ^{1}H-NMR spectra of *N*-(3-(ethyldisulfanyl)-5-hydroxypent-2-en-2-yl)-*N*-methylformamide 5a ($CDCl_3$, 400MHz).

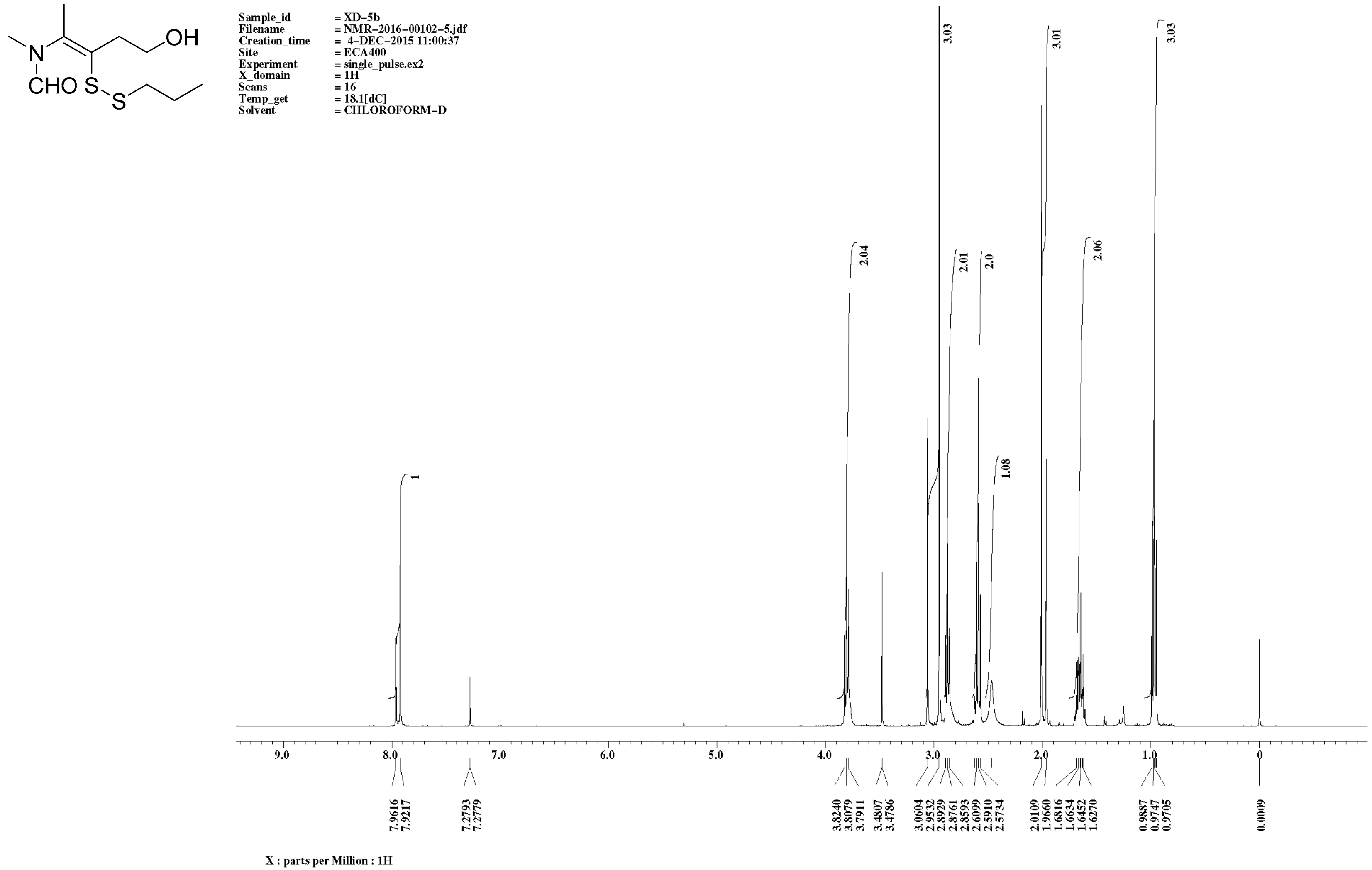

Figure S2. ^{1}H-NMR spectra of *N*-(3-(propyldisulfanyl)-5-hydroxypent-2-en-2-yl)-*N*-methylformamide 5b ($CDCl_3$, 400MHz).

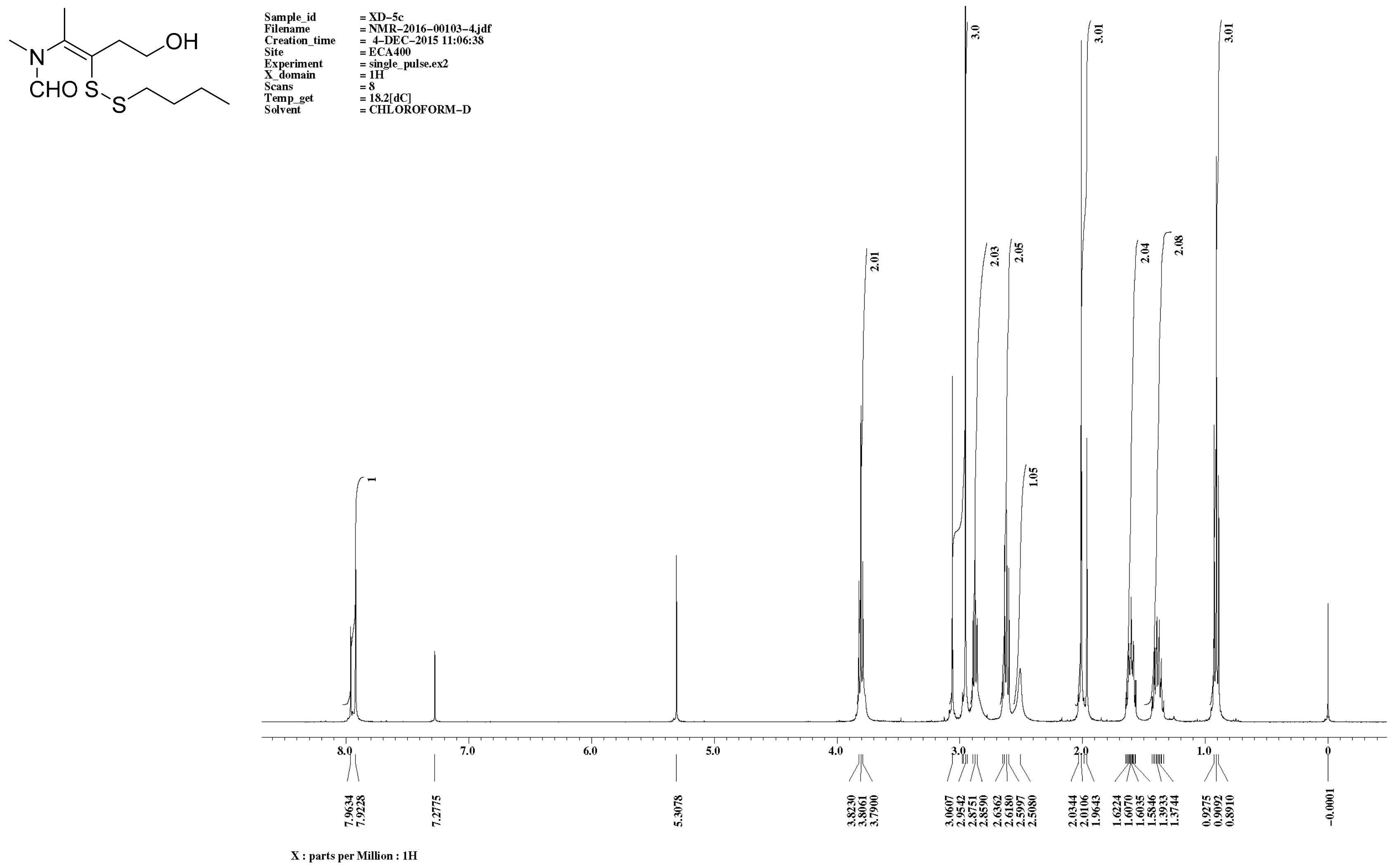

Figure S3. ^{1}H-NMR spectra of *N*-(3-(butyldisulfanyl)-5-hydroxypent-2-en-2-yl)-*N*-methylformamide 5c ($CDCl_3$, 400MHz).

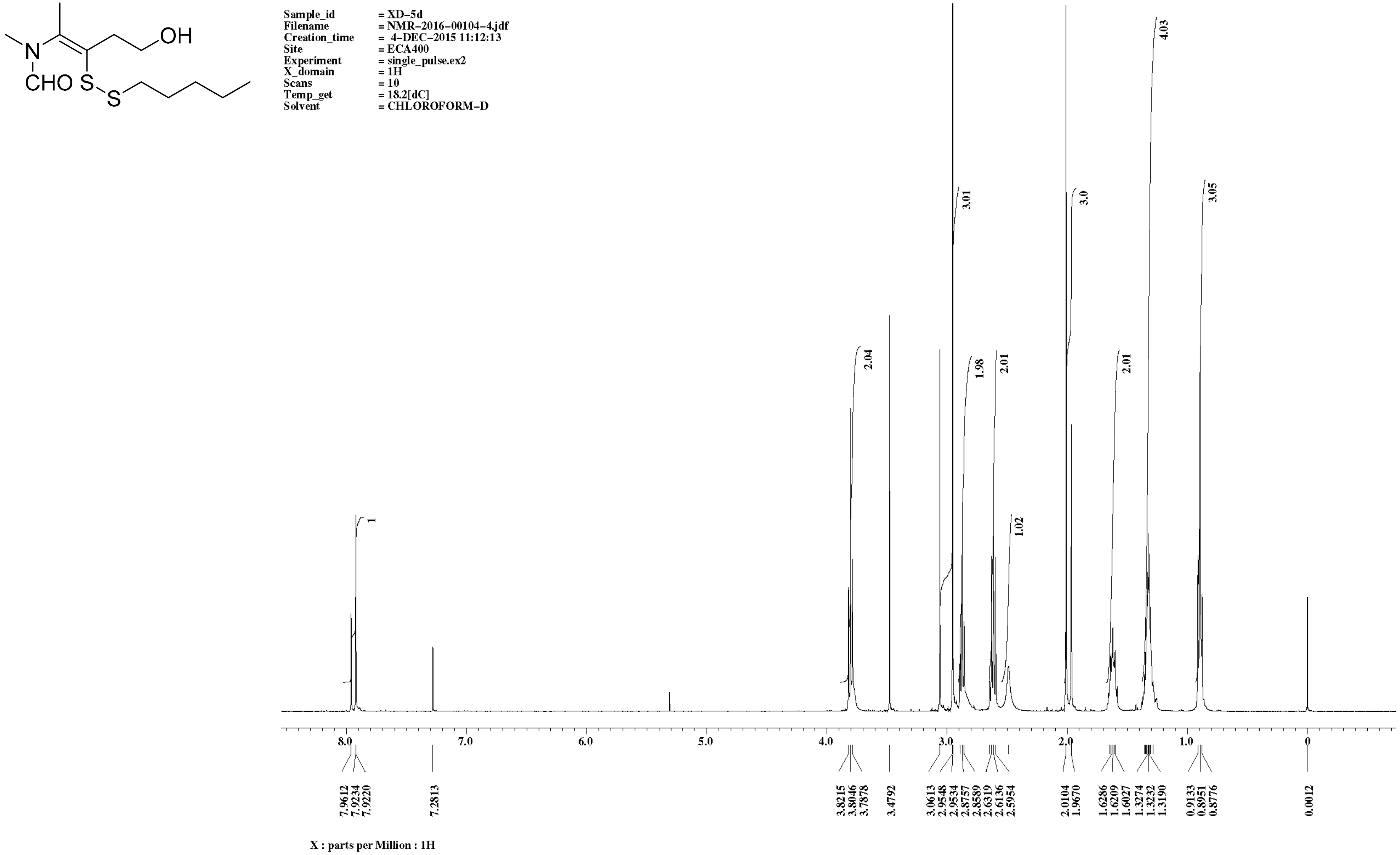

Figure S4. ^{1}H-NMR spectra of *N*-(3-(amyldisulfanyl)-5-hydroxypent-2-en-2-yl)-*N*-methylformamide 5d ($CDCl_3$, 400MHz).

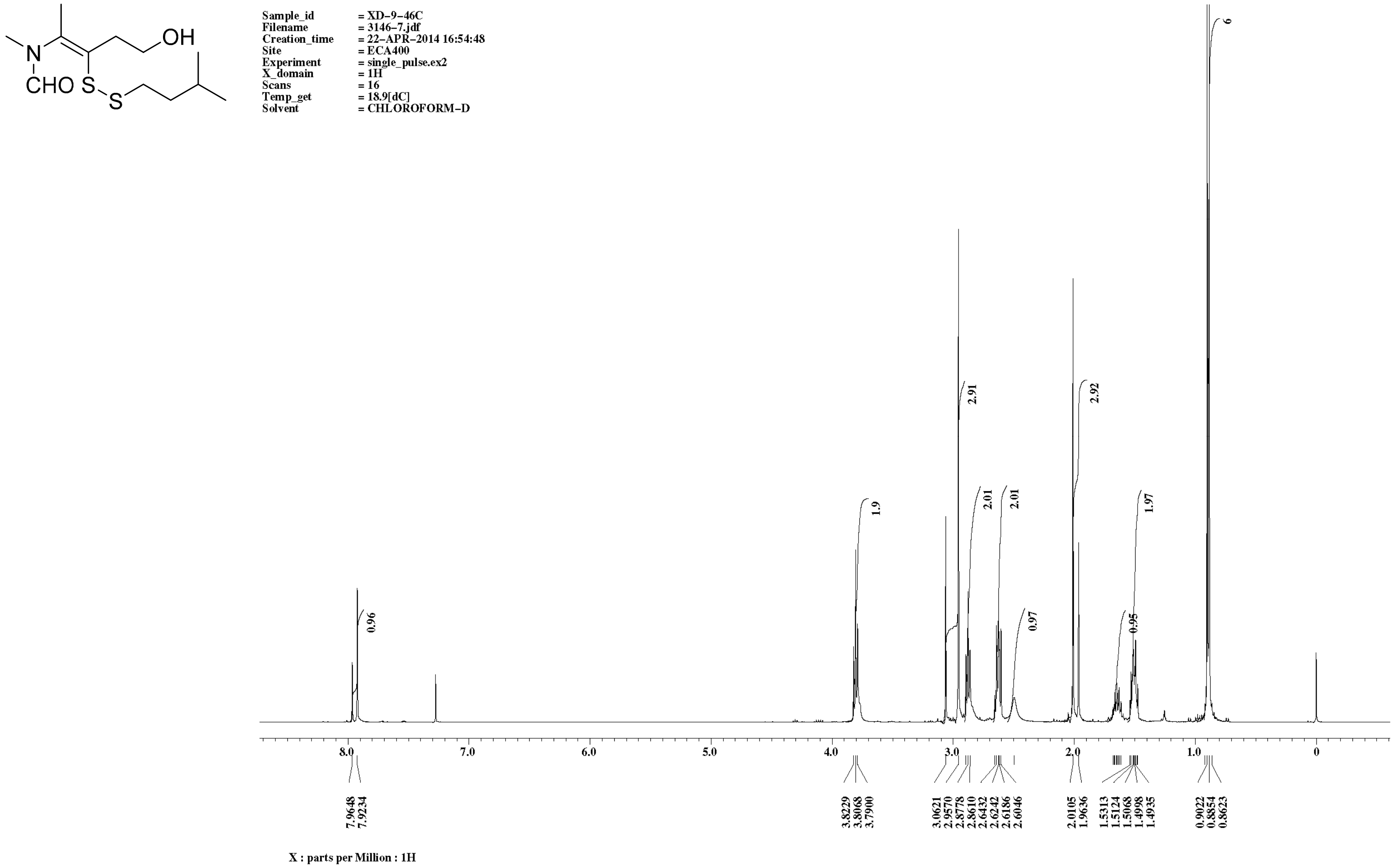

Figure S5. ^{1}H-NMR spectra of *N*-(3-(isoamyldisulfanyl)-5-hydroxypent-2-en-2-yl)-*N*-methylformamide 5e ($CDCl_3$, 400MHz).

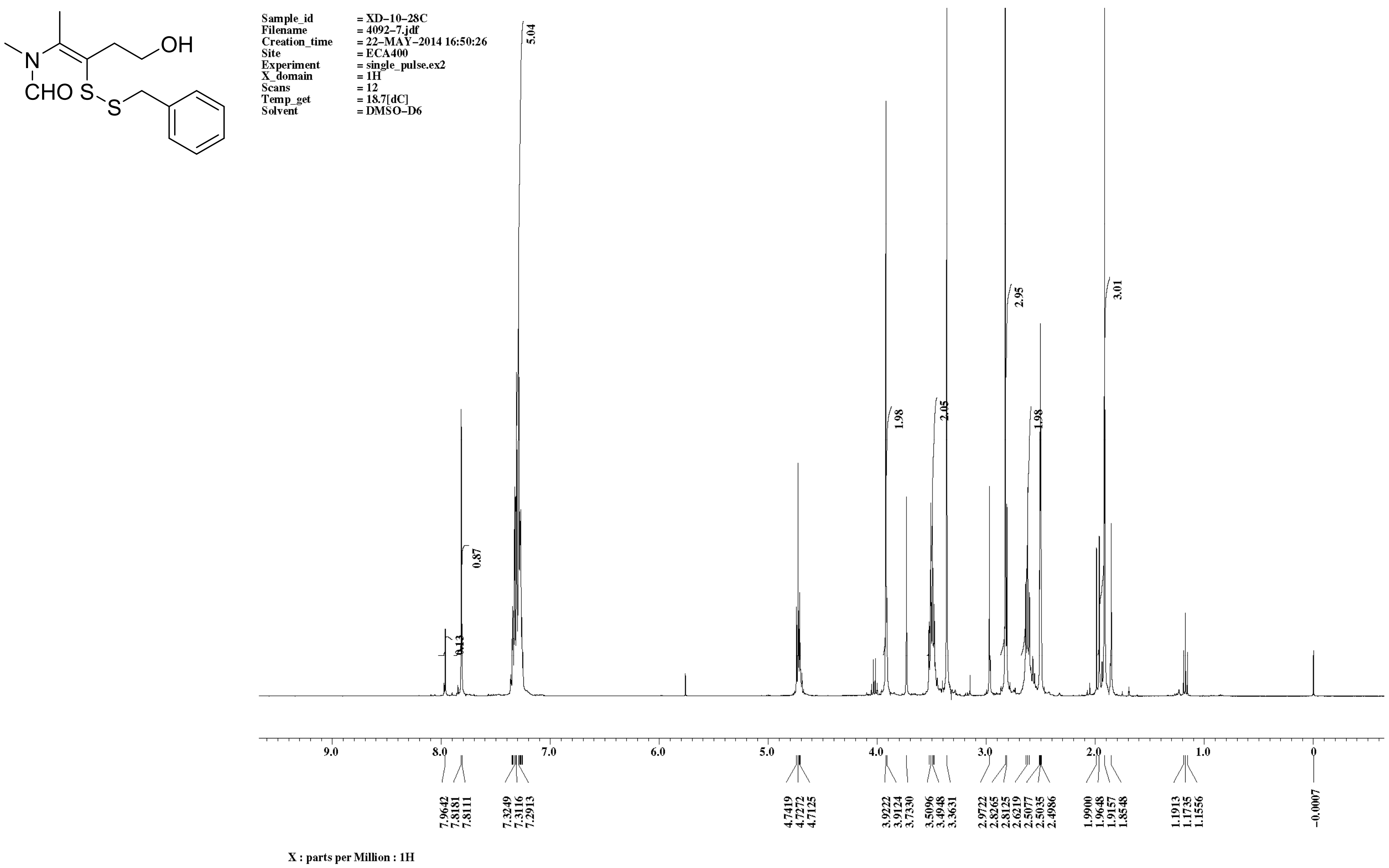

Figure S6. ^{1}H-NMR spectra of *N*-(3-(benzyldisulfanyl)-5-hydroxypent-2-en-2-yl)-*N*-methylformamide 5f (DMSO-d_6, 400MHz).

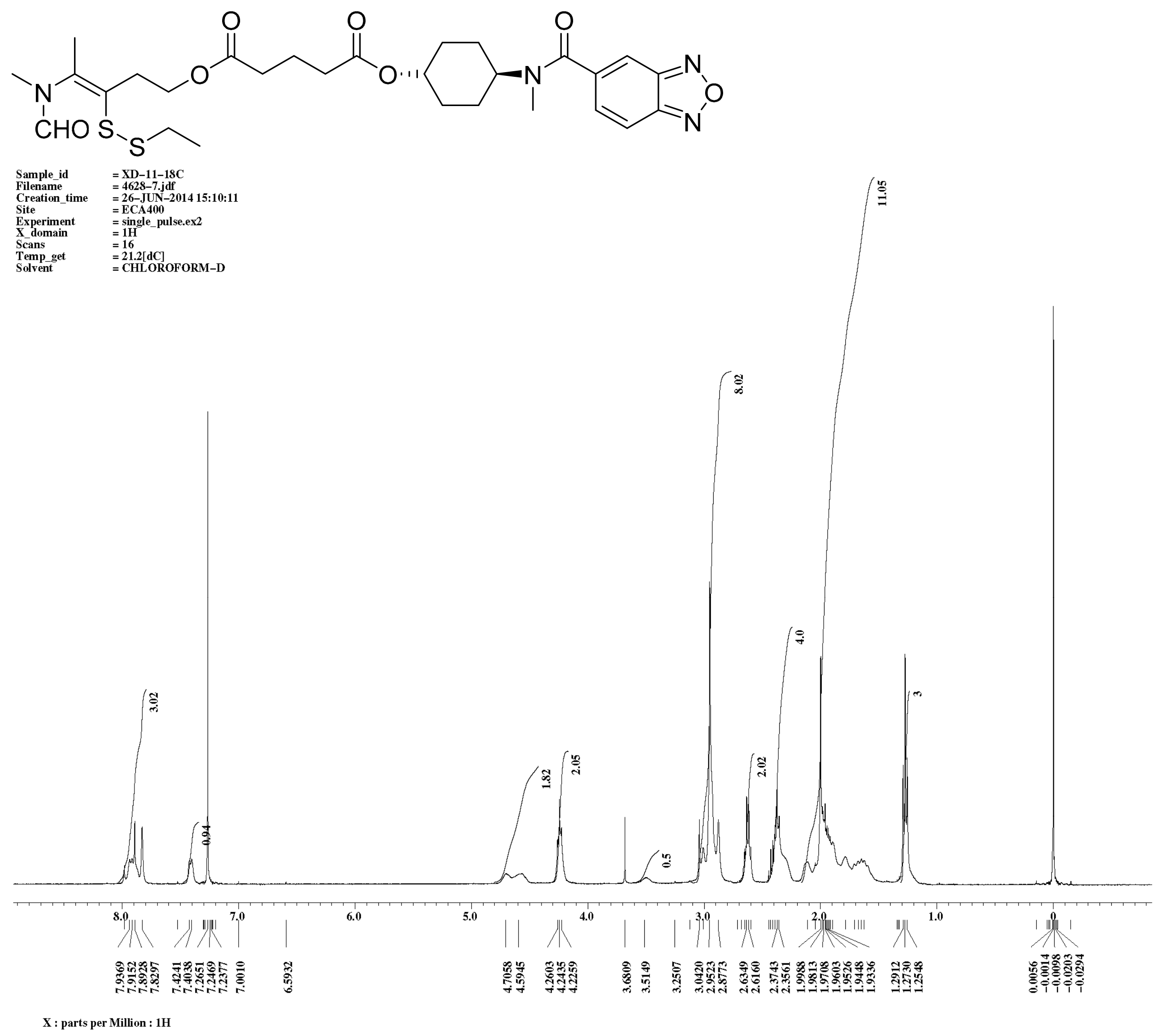

Figure S7. ^{1}H-NMR spectra of *S*-3-(ethyldisulfanyl)-4-(*N*-methylformamido)pent-3-en-1-yl((1R,4R)-4-(*N*-methylbenzo[c][1,2,5]oxadiazole-5-carboxamido)cyclohexyl) glutarate (**7a**) ($CDCl_3$, 400MHz).

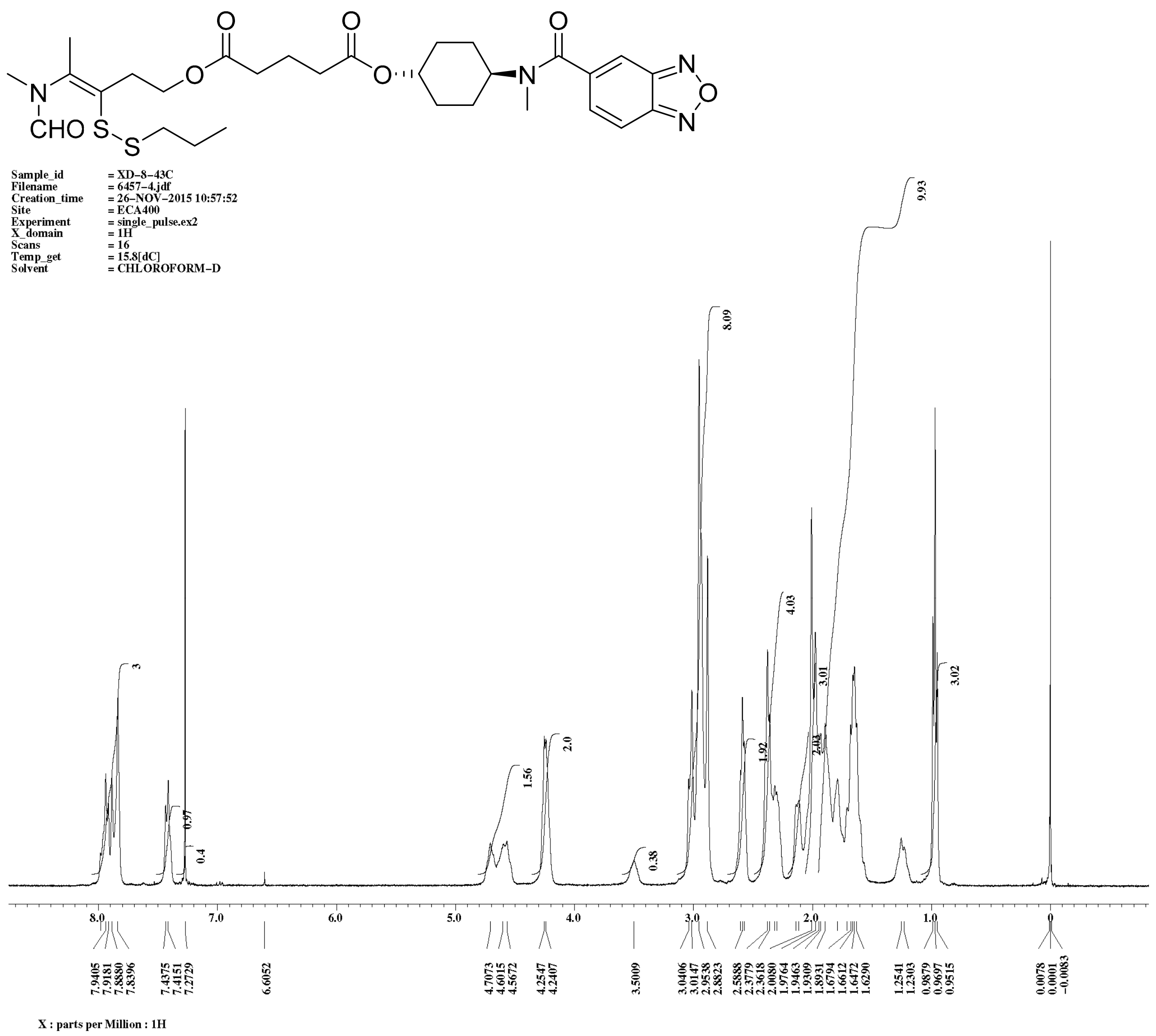

Figure S8. ^{1}H-NMR spectra of *S*-3-(propyldisulfanyl)-4-(*N*-methylformamido)pent-3-en-1-yl((1R,4R)-4-(*N*-methylbenzo[c][1,2,5]oxadiazo-le-5-carboxamido)cyclohexyl) glutarate (**7b**) ($CDCl_3$, 400MHz).

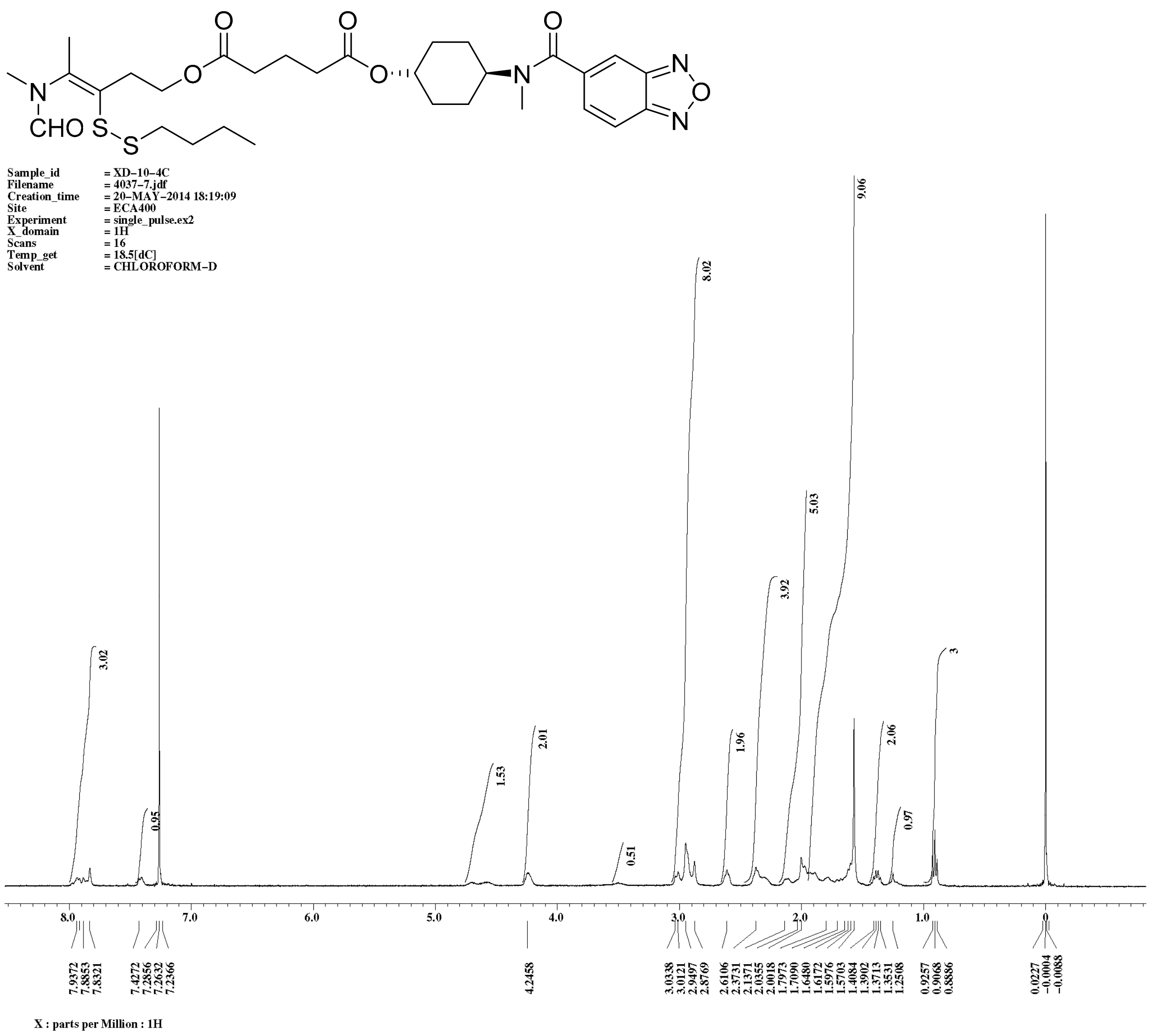

Figure S9. ^{1}H-NMR spectra of *S*-3-(butyldisulfanyl)-4-(*N*-methylformamido)pent-3-en-1-yl((1R,4R)-4-(*N*-methylbenzo[c][1,2,5]oxadiazole-5-carboxamido)cyclohexyl) glutarate (**7b**) ($CDCl_3$, 400MHz).

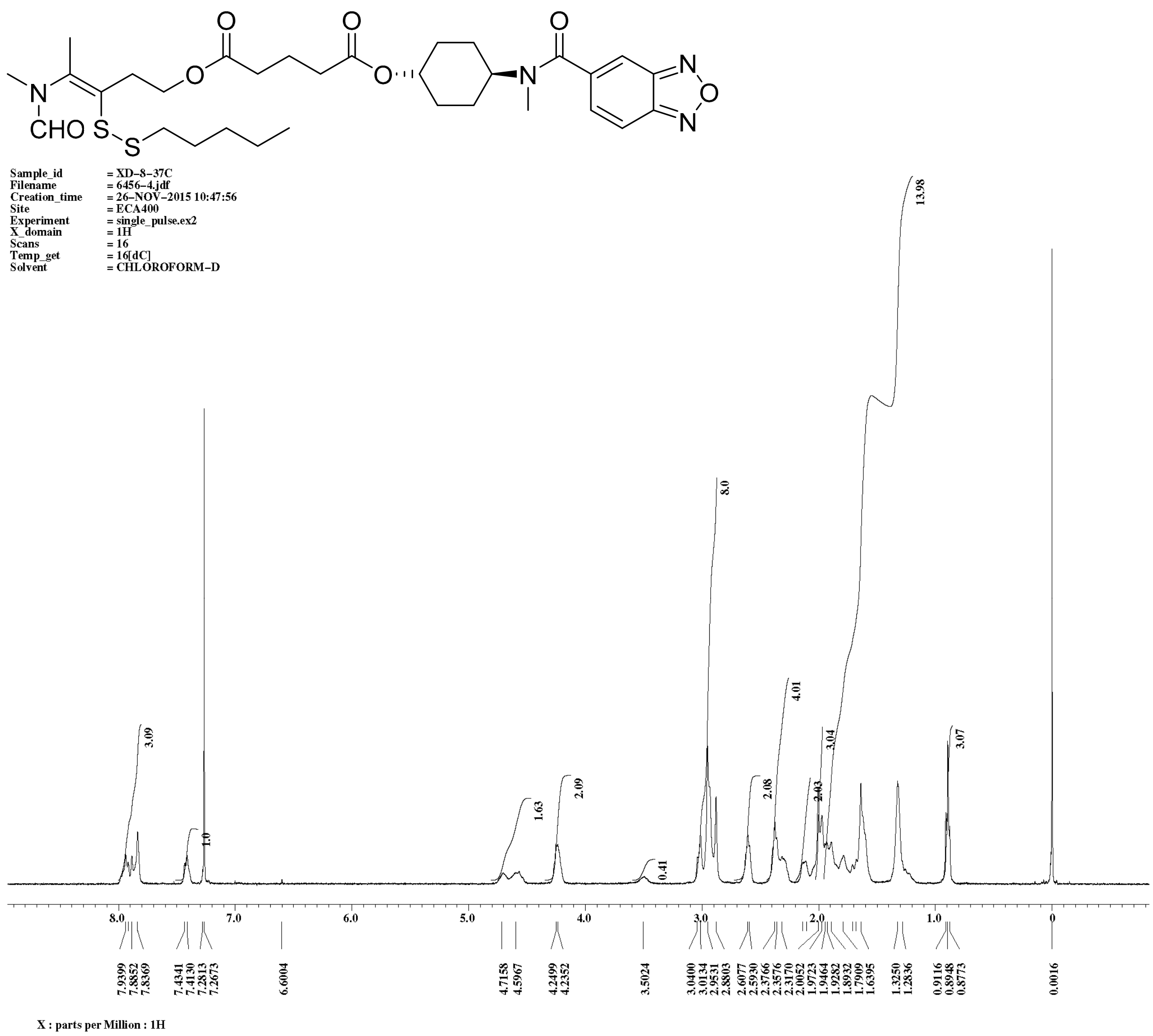

Figure S10. ^{1}H-NMR spectra of *S*-3-(amyldisulfanyl)-4-(*N*-methylformamido)pent-3-en-1-yl((1R,4R)-4-(*N*-methylbenzo[c][1,2,5]oxadiazole-5-carboxamido)cyclohexyl) glutarate (**7d**) ($CDCl_3$, 400MHz).

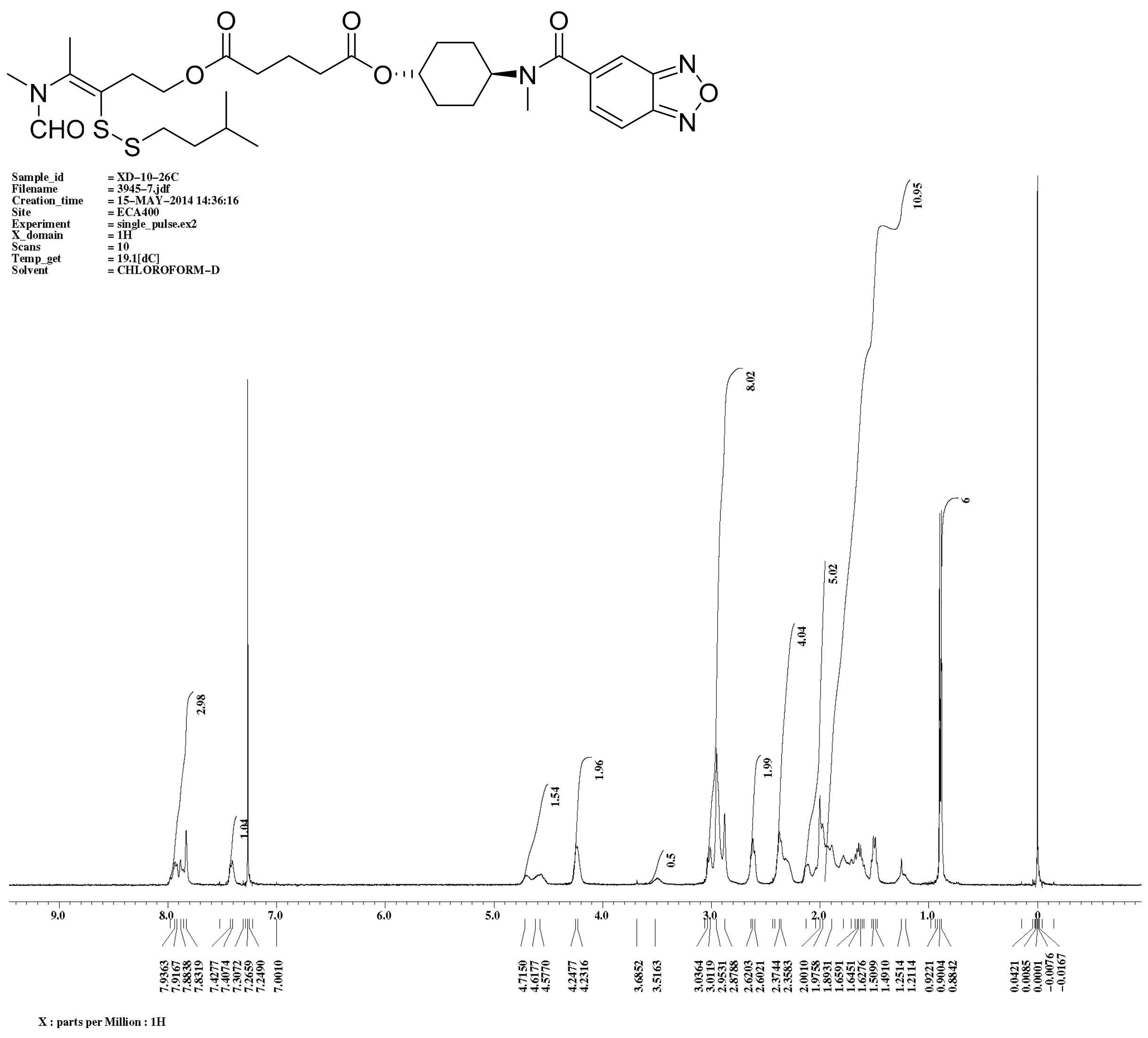

Figure S11. ^{1}H-NMR spectra of *S*-3-(isoamyldisulfanyl)-4-(*N*-methylformamido)pent-3-en-1-yl((1R,4R)-4 -(*N*-methylbenzo[c][1,2,5]oxadia-zole-5-carboxamido)cyclohexyl) glutarate (**7d**) ($CDCl_3$, 400MHz).

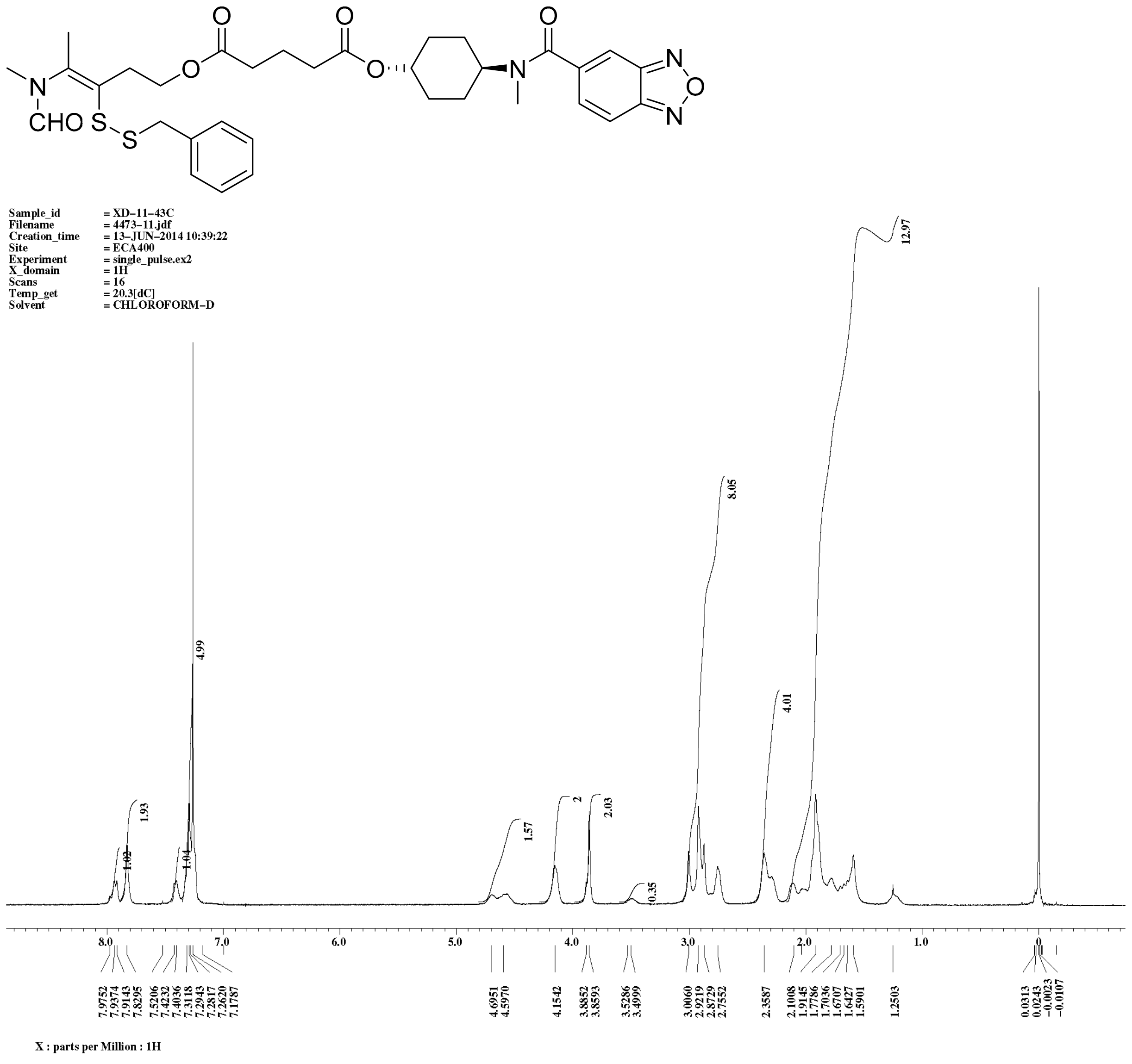

Figure S12. ^{1}H-NMR spectra of *S*-3-(benzyldisulfanyl)-4-(*N*-methylformamido)pent-3-en-1-yl((1R,4R)-4-(*N*-methylbenzo[c][1,2,5]oxadia-z-ole-5-carboxamido)cyclohexyl) glutarate (**7d**) ($CDCl_3$, 400MHz).
